# Next generation bioelectronic medicine: making the case for non-invasive closed-loop autonomic neuromodulation

**DOI:** 10.1186/s42234-024-00163-4

**Published:** 2025-01-21

**Authors:** Imanuel Lerman, Yifeng Bu, Rahul Singh, Harold A. Silverman, Anuj Bhardwaj, Alex J. Mann, Alik Widge, Joseph Palin, Christopher Puleo, Hubert Lim

**Affiliations:** 1 Department of Electrical and Computer Engineering, University of California San Diego, Atkinson Hall, 3195 Voigt Dr., La Jolla, CA 92093 USA; 2 Center for Stress and Mental Health (CESAMH) VA San Diego, La Jolla, CA 92093 USA; 3 Department of Anesthesiology, University of California San Diego, La Jolla, CA 92093 USA; 4 InflammaSense Incorporated Head Quarters, La Jolla, CA 92093 USA; 5 Wolf Greenfield Biotechnology Practice Group, New York, NY 10158 USA; 6 SecondWave Systems Incorporated, Head Quarters, Minneapolis-Saint Paul, MN 55104 USA; 7 hVIVO Limited, Head Quarters, London, E14 5NR UK; 8 Department of Psychiatry & Behavioral Sciences, University of Minnesota, Minneapolis, MN 55454 USA; 9 Convergent Research Inc, Head Quarters, Cambridge, MA 02138-1121 USA; 10 Department of Biomedical Engineering, Center for Biotechnology and Interdisciplinary Studies, Rensselaer Polytechnic Institute, Rensselaer, NY 12180 USA; 11 Department of Biomedical Engineering, University of Minnesota, Minneapolis, MN 55455 USA; 12 Department of Otolaryngology, University of Minnesota, Minneapolis, MN 55455 USA

**Keywords:** Closed loop bioelectronic medicine, Neuromodulation, Bioelectronic medicine, Focused ultrasound stimulation, Autonomic neurography, Neurography, Vagus nerve

## Abstract

The field of bioelectronic medicine has advanced rapidly from rudimentary electrical therapies to cutting-edge closed-loop systems that integrate real-time physiological monitoring with adaptive neuromodulation. Early innovations, such as cardiac pacemakers and deep brain stimulation, paved the way for these sophisticated technologies. This review traces the historical and technological progression of bioelectronic medicine, culminating in the emerging potential of closed-loop devices for multiple disorders of the brain and body. We emphasize both invasive techniques, such as implantable devices for brain, spinal cord and autonomic regulation, while we introduce new prospects for non-invasive neuromodulation, including focused ultrasound and newly developed autonomic neurography enabling precise detection and titration of inflammatory immune responses. The case for closed-loop non-invasive autonomic neuromodulation (incorporating autonomic neurography and splenic focused ultrasound stimulation) is presented through its applications in conditions such as sepsis and chronic inflammation, illustrating its capacity to revolutionize personalized healthcare. Today, invasive or non-invasive closed-loop systems have yet to be developed that dynamically modulate autonomic nervous system function by responding to real-time physiological and molecular signals; it represents a transformative approach to therapeutic interventions and major opportunity by which the bioelectronic field may advance. Knowledge gaps remain and likely contribute to the lack of available closed loop autonomic neuromodulation systems, namely, (1) significant exogenous and endogenous noise that must be filtered out, (2) potential drift in the signal due to temporal change in disease severity and/or therapy induced neuroplasticity, and (3) confounding effects of exogenous therapies (e.g., concurrent medications that dysregulate autonomic nervous system functions). Leveraging continuous feedback and real-time adjustments may overcome many of these barriers, and these next generation systems have the potential to stand at the forefront of precision medicine, offering new avenues for individualized and adaptive treatment.

## Background

Bioelectronic medicine has deep historical roots, with records dating back to ancient Egypt, where electric fish were used to deliver therapeutic shocks for conditions such as headaches—the first documented instance of non-invasive neuromodulation (Kellaway 1946). Early Greek physicians applied similar treatments for ailments such as gout and arthritis (Zubairi 2023). However, the field began its evolution toward modern bioelectronic medicine in the late eighteenth century with Luigi Galvani’s pioneering experiments demonstrating muscle contraction in frog legs via electrical stimulation (Whittaker 1954). Alessandro Volta’s development of the battery soon followed, sparking interest in using electricity therapeutically for paralysis and pain relief. A pivotal breakthrough came in the late nineteenth century with the advent of heart monitoring. Augustus Waller’s use of a capillary electrometer to capture a crude heartbeat signal (Luderitz 2003), and Willem Einthoven’s refinement of this technology with his string galvanometer to produce the PQRST waveform, marked the beginning of a new era in bioelectronic medicine (Hurst 1998).

The field saw exponential growth from the 1950s to the 2000s, underpinned by advancements in cardiac pacemakers, deep brain stimulation (DBS), spinal cord stimulation (SCS), peripheral prosthetics, and wearable health technologies. These innovations not only revolutionized therapeutic interventions but also established the foundation for closed-loop bioelectronic systems, particularly in the modulation of the autonomic nervous system. Today, both invasive and non-invasive approaches leverage real-time physiological data to provide dynamic, adaptive treatments for a wide array of conditions, including neuroimmune disorders. This trajectory highlights the immense potential of bioelectronic medicine to transform personalized healthcare through precision-targeted, closed-loop therapies leveraging innovations in physiological and molecular sensors.

### The evolution of pacemakers, deep brain stimulators, and spinal cord stimulators: a construct for non-invasive closed loop bioelectronic medicine

The history of cardiac pacing is deeply intertwined with the evolution of bioelectronic medicine, reflecting pivotal milestones that transformed our understanding of how external electrical stimulation can control biological processes. Early attempts to stimulate the heart with electricity date back to the eighteenth century, with Luigi Galvani's discovery of bioelectricity in 1791, which demonstrated the ability to contract muscles using electrical stimulation (Whittaker 1954). However, the advent of pacemakers in the mid-twentieth century marked a significant leap in applying electrical stimulation for therapeutic purposes.

The development of modern pacemakers began in the 1950s, when external devices powered by wall outlets were first used to maintain heart rhythms in patients with heart block. One of the first significant innovations came from Paul Zoll, a pioneer who developed an external tabletop pacemaker in 1952 (Mahapatra 2021; Nelson 1993). His device, though revolutionary, was bulky, uncomfortable for patients due to its high-voltage shocks, and reliant on external power, which limited its practicality. A critical breakthrough occurred in 1957 when engineer Earl Bakken and surgeon C. Walton Lillehei at the University of Minnesota introduced the first battery-powered, portable pacemaker (Mahapatra 2021; Nelson 1993). This device allowed for continuous cardiac pacing post-surgery and offered a significant improvement in patient mobility and safety; it was the first step in miniaturization and toward wearable or implantable systems. Bakken's innovation was a precursor to fully implantable pacemakers, which were developed shortly thereafter by Ake Senning and Rune Elmqvist in 1958. Their first successful implantation in a human marked the beginning of an era in which patients with complete heart block could receive reliable, long-term pacing support (Mahapatra 2021), in a fully implantable solution in which the patient and system were completely mobile.

The transition from these early pacemakers to modern, closed-loop systems underscores the technological evolution within bioelectronic medicine. Early pacemakers were fixed-rate devices, incapable of responding to the physiological needs of the patient. However, advancements in the 1970s and 1980s introduced rate-responsive pacemakers that could adapt to the patient's activity level, improving both quality of life and survival outcomes. By integrating feedback mechanisms, modern pacemakers now function within a closed-loop system, where they sense physiological parameters and adjust their output in real time, thereby mimicking natural cardiac function more effectively.

The same principles that revolutionized cardiac pacing—such as the integration of sensors, real-time data processing, and feedback loops—are now being applied to broader areas of bioelectronic medicine. The evolution from simple open-loop systems to sophisticated closed-loop devices underscores the potential for bioelectronic medicine to move beyond heart rhythm management (Ghanim 2023). With advances in sensor technology and computational power, bioelectronic devices are increasingly capable of detecting physiological changes related to inflammation, immune responses, and other pathologies, offering targeted, real-time therapeutic interventions. Innovations in molecular sensors, such as for detecting glucose, hormones or cytokines, are also emerging as wearable and at-home technologies. The rise of wearable bioelectronic devices also began in the late twentieth century. From early heart rate monitors in the 1970s (Holter 1961) to wearable defibrillators by the 2000s (Bardy et al. 2010), these technologies became a vital part of health monitoring.

In the 1980s, deep brain stimulation (DBS) emerged as a revolutionary therapeutic option for patients with movement disorders, particularly Parkinson's disease (Benabid et al. 1987). DBS was developed as a more flexible alternative to irreversible lesioning techniques, offering adjustable, reversible neuromodulation (Lozano and Lipsman 2013). In 1997, the U.S. Food and Drug Administration (FDA) approved the Medtronic DBS system for Essential Tremor and in 2003 followed with a second approval for Parkinson’s disease, solidifying its role as a critical advancement in bioelectronic medicine (Regulatory n.d). These systems provided remarkable capability for the neurologist physician, where within 5–10 min they could initiate a therapy program through a remote control that resulted in instantaneous cessation of the bothersome tremor that plagued an Essential Tremor or Parkinson’s disease patient. Following these device successes, Medtronic’s DBS System for epilepsy was FDA approved in 2018 for adults with focal epilepsy who did not respond to at least three anti-seizure medications (Regualtoery n.d.). The late 1990s and early 2000s saw the development of closed-loop DBS systems, which adjust stimulation in response to real-time neural signals, representing an important step towards more precise neuromodulation technologies (Rosin et al. 2011). However, the technology was not approved for clinical use in Parkinson’s disease until 2020 with Medtronic’s Percept™ with Brainsense™ DBS system (Cuschieri et al. 2022). Similarly, the period from the 1960s to 2000s was marked by significant advancements in spinal cord stimulation (SCS), particularly for pain management (Caylor et al. 2019) and motor function restoration in patients with spinal cord injuries (SCI). Early SCS systems focused on modulating pain pathways, while later innovations, particularly in the 1990s, incorporated more advanced neurostimulation techniques aimed at assisting motor recovery. By the early 2000s, closed-loop SCS systems were being developed, which modulate electrical signals based on real-time physiological responses, enhancing the therapeutic potential of SCS for both pain management and motor function recovery. Similar to DBS systems, Saluda and Medtronic now have developed closed loop SCS systems recently FDA approved (2022 and 2024, respectively) for pain management while Onward Medical has recently applied for the first DeNovo closed-loop SCS system aimed to treat SCI (Team 2024). While no closed loop devices have yet to be developed, the late 1990s saw the emergence of bioelectronic approaches to modulating the autonomic nervous system (ANS), with vagus nerve stimulation (VNS) leading the charge. Initially developed as a treatment for epilepsy and major depression disorder (Ben-Menachem et al. 2015), surgically implanted (minimally invasive) VNS was later investigated for its ability to regulate immune responses and inflammation, marking a significant shift towards using bioelectronic medicine for pathologies not traditionally viewed as a neural disorder, such as rheumatoid arthritis (Koopman et al. 2016a). This VNS work laid the foundation for the broader application of bioelectronic devices in treating inflammation and immune-related conditions (Koopman et al. 2016a; Pavlov and Tracey 2017).

### Non-invasive closed-loop bioelectronic medicine

A non-invasive procedure colloquially does not require any incisions or penetration of the body. It typically involves external techniques such as applying external devices, imaging, or treatments that do not physically enter the body. A minimally invasive procedure requires small incisions or minor interventions that enter the body, but with minimal trauma to tissues. In contrast, invasive procedures involve significant penetration into the body, often requiring larger incisions or deep insertion of instruments or devices into tissues, organs, or body cavities. These types of procedures generally result in more tissue damage, longer recovery times, and higher risk of complications. As an exemplar, non-invasive closed loop wearable devices have been developed to treat cerebral palsy related foot drop wherein neural stimulation is synced to foot acceleration allowing for significant improvement in gait (Robison 2027; Pool et al. 2015), in which no component of the system is implanted. Non-invasive wearable devices have similarly shown efficacy and are now approved for essential tremor (Dai et al. 2023). Advancements in prosthetics also experienced rapid growth during this period, largely driven by the integration of bioelectronic interfaces. Minimally invasive myoelectric prosthetics, which use muscle signals to control limb movement, began gaining traction in the 1960s. Over the next few decades, these systems became increasingly sophisticated, incorporating neural control mechanisms that enabled more intuitive and precise control over artificial limbs that has resulted in FDA-approved devices such as the DEKA arm in 2014 (Currents 2014). The advent of invasive brain-computer interface (BCI) technology in the early 2000s further accelerated this field, allowing for more direct neural input to prosthetic devices, rapidly translated from pre-clinical non-human primates to human use which improved functionality and user satisfaction (Heller et al. 1991; Cordella et al. 2016; Lebedev and Nicolelis 2017; Ciancio et al. 2016). More recently, noninvasive hybrid systems have been deployed in which brain signals are transmitted to a non-invasive high spatial resolution neurostimulation arm sleeve-based device allowing for the user to carry out specific tasks; in a memorable case, a fully paralyzed patient was able to learn and play a Nintendo Guitar Hero video game enabled by the BCI coupled to the neural sleeve (Sharma et al. 2016).

Additionally, transcranial magnetic stimulation (TMS), which was developed in the mid-1980s (Barker et al. 1985), emerged as a non-invasive bioelectronic method for modulating brain activity.

Initially applied for diagnostic purposes, TMS was later explored for therapeutic uses, especially in treating depression. In 2008, the FDA approved TMS for depression (O’Reardon et al. 2007), signifying its importance in both clinical and research settings. Recent trials of non-invasive closed-loop TMS have shown variable efficacy across different neurological and psychiatric conditions. In depression, Karabanov et al. (2016) reported promising results with real-time adjustment of stimulation parameters (Karabanov et al. 2016). Studies like the Stanford Accelerated Intelligent Neuromodulation Therapy (SAINT) demonstrated an 80% remission rate in depression through real-time brain monitoring and adaptive stimulation (Cole et al. 2022). In stroke rehabilitation, closed loop TMS investigators have observed enhanced neuroplasticity and motor function recovery (Singh et al. 2023; Gharabaghi et al. 2014). While these studies demonstrate potential, efficacy varies across conditions and individuals; effects are influenced by heterogenous factors such as brain anatomy, disease severity and optimal stimulation parameters. The future success of TMS will need to leverage more sophisticated and precise closed-loop TMS therapy, such as leveraging EEG temporal dynamics and brain states with multi-scale modeling, which could improve the treatment for multiple brain disorders (Wischnewski 2024; Shirinpour et al. 2021). Larger clinical trials are also necessary to fully establish the effectiveness of non-invasive closed-loop TMS across various disease disorders that will allow improved patient selection and treatment outcomes.

**Fig. 1:**
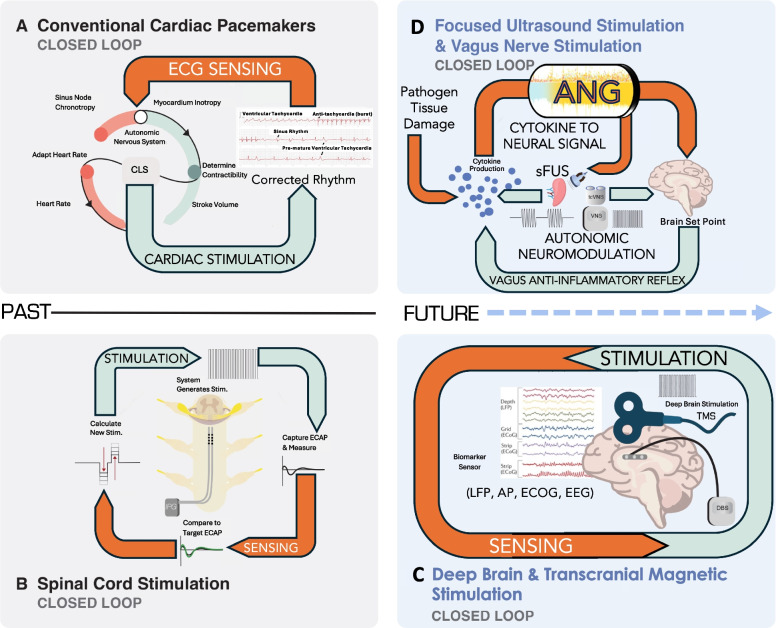
Closed Loop Neuromodulation/Cardiac Devices: input a signal that modulates the output of the neuromodulation target (Dark Orange Arrows = sensing, Light Green Arrows = Stimulation). By definition, closed loop neuromodulation requires a continuous sensing modality that then continuously modifies the stimulation parameter. Autonomic Neuromodulation may include invasive electrical neural stimulation, as well as non-invasive electrical (e.g., transcutaneous vagus nerve stimulation (tcVNS)] and directed energy devices that contribute to autonomic nervous system modulation effects, (e.g., with splenic focused ultrasound stimulation (sFUS)). Current devices on the market include conventional cardiac pacemakers (Diack et al. 1979) (**A**), and Closed Loop Evoked Compound Action Potential (ECAP) spinal cord stimulation (SCS) marketed for pain (Caylor et al. 2019) (**B**). Recent advances in brain recording have resulted in development of Close Loop Deep Brain Stimulation (DBS) and Closed Loop Transcranial Magnetic Stimulation (TMS) (**C**). Closed Loop DBS is now FDA approved for Parkinson’s Disease (PD) and epilepsy, while other disease states are currently being studied (e.g., PTSD or Depression (Widge 2023), Pain (Shirvalkar et al. 2018), and Alzheimer's Disease (Ríos et al. 2022; Hell et al. 2019)). Future Closed Loop Autonomic Neuromodulation (**D**) is under development in which inflammation is sensed with Autonomic Neurography (ANG) that can then drive autonomic neuromodulation delivered or to hone in on the dosage, aimed to amplify the body’s own vagus-driven anti-inflammatory reflex, thus throttling down inflammation by regulating macrophages that circulate through the spleen

Defibrillation, first conceptualized in 1898, advanced significantly with the creation of the first implantable pacemaker in 1958 and the development of fully automated external defibrillators by the late 1970s (Altman and Arne 2002). These devices could autonomously analyze and respond to heart rhythms. Over the course of nearly six decades, researchers brought together diagnostic and therapeutic technologies to create fully integrated closed-loop systems capable of proactively monitoring and maintaining patient health (Diack et al. 1979). While invasive closed-loop bioelectronic therapies have faced extended timelines for regulatory approval, the development of non-invasive technologies has accelerated. Non-invasive systems benefit from reduced preclinical, clinical, and regulatory hurdles, enabling faster innovation and widespread availability. Despite these advances, there is still a need for minimally invasive and invasive systems. Non-invasive technologies often face limitations compared to invasive systems in terms of precision of their temporal and spatial resolution, both in recording and neuromodulation. As bioelectronic medicine continues to evolve, both invasive and non-invasive approaches will need to advance in parallel to provide tailored, high-precision therapies that meet the unique needs of individual patients Fig. 1.

The review of past closed loop neuromodulation or cardiac devices provides the necessary context to understand the evolution of the next generation of non-invasive closed loop bioelectronic medicine, particularly in relation to the development from invasive to minimally invasive and now towards non-invasive closed loop bioelectronic medicine systems. Collectively, by emphasizing key past milestones in pacemakers, DBS, SCS, prosthetics, wearable technologies, TMS, and ANS neuromodulation, we aim to underscore how these core innovations have shaped the modern landscape and provide a road map for future development of non-invasive closed loop bioelectronic medicine technologies.

## Main text

### Modern era of non-invasive bioelectronic medicine

Compared to the experience with pacemakers and automated external defibrillators, there has recently been a rapid expansion of bioelectronic medicine from foundational science and diagnostics to therapeutics with systems approaching closed loop implementation with commercially viable technologies. In the 2010s, National Institutes of Health (NIH) pushed the field of bioelectronic medicine ahead at an accelerating pace with the NIH Stimulating Peripheral Activity to Relieve Conditions (SPARC) program to map, characterize, and understand how the human nervous system regulates and responds to organ function. The SPARC program is enabling discoveries in the realm of anatomy, physiology, diagnostics, and therapeutics all in parallel (Qashu 2024). For example, recent work from the SPARC program has brought forward the Center for Autonomic Nerve Recording and Stimulation Systems (CARSS) team; they are developing open-source solutions for recording and stimulating peripheral nerves (Pikov n.d.), highlighting the field is moving toward funding these closed loop solutions.

In conjunction with NIH SPARC efforts, Defense Advanced Research Projects Agency (DARPA) accelerated invasive neuromodulation technology with Reliable Neural-Interface Technology (RE-NET), minimally invasive bioelectronic medicine with Next-Generation Nonsurgical Neurotechnology (N^3^) program and both invasive and non-invasive Electrical Prescriptions (ElectRx) programs. The ElectRx program was aimed to advance the understanding of the peripheral nervous system as well as supporting novel invasive and noninvasive technologies for treating various acute and chronic immune disorders and infectious diseases (Naufel et al. 2020). Equivalent to NIH SPARC CARSS work, ElectRx aimed to develop closed-loop neuromodulation-based solutions (both minimally invasive and non-invasive) that continuously calibrated the therapy based on defined physiological and molecular biomarkers. Both the DARPA and SPARC programs were based on the premise that pharmacological interventions are incomplete on their own, while all pharmacological interventions result in off-target systemic side effects. Regrettably, current pharmacological regulatory approval processes incentivize non-precision expedited go-to-market drug/device development, resulting in inadequate drug and device parameter selection; it can result in an incomplete drug or device treatment titration for each individual’s specific needs (Tyson et al. 2020). Relegated to single randomized controlled clinical trial standards, core understanding that can chart how different illnesses manifest and spread across a specific individual’s organs over time (beyond the period of a particular clinical trial) are largely ignored in the existing drug and device approval paradigms. Unfortunately, there are large gaps in our understanding of how certain disease states directly or indirectly modulate central nervous and peripheral nervous system (CNS, PNS) function; it presents an unmet need in which our field may develop treatments to correct disease mediated co-morbid CNS and/or PNS dysfunction. Teleologically, targeted neuromodulation may fill this treatment gap by correcting CNS and PNS dysfunction, however most tools available are uninformed on the dose needed. As an exemplar, in some patients the immune response may be excessive or over-responsive causing harm to the patient, while in other patients a blunted immune response can lead to morbidity or mortality; but in both cases and across the spectrum, closed-loop autonomic neuromodulation may provide individualized titration of the treatment to the desired effect.

### Cutting edge developments

#### Diagnostics—sensing

Research successes out of the NIH and DARPA programs have paved the way to a paradigm changing landscape for both diagnosis and treatment by using advanced bioelectronic medicine devices. In the BCI prosthetic field, rapid advancements have been realized (Nicolelis 2003) where recording and decoding neuronal ensemble activity for robotic arm control has been demonstrated in preclinical and now routinely in human subjects (Collinger et al. 2013; Sitaram et al. 2017). Research groups strove to maximize implantable electrode spatial resolution thereby improving algorithms, while recent minimally invasive neurovascular electrode recording methodologies coupled to the rapid advancement in machine and deep learning algorithms may in some cases circumvent the need for high spatial resolution (Oxley et al. 2021). These minimally invasive electrode devices (i.e. recently developed Synchron’s Stentrode™) leverage machine and deep learning (ML and DL) approaches that can distinguish specific feature importance that in real time reliably cypher the neural code into actionable prosthetic activities (Forsyth 2019; Oxley and Opie 2018).Leveraging these ML and DL approaches to decode certain autonomic neuronal responses acquired with lower spatial resolution non-invasive electrode arrays may also circumvent the need for high spatial resolution penetrating electrode arrays.

Beyond brain machine interfaces that decode the thoughts or intentions, peripheral neuronal machine interfaces are now capable of detecting the neuronal signal from the body to the brain and decoding the information being sent in real time (Ding et al. 2024). These body to brain signals are integrated providing a richer sense of the change in the body response to exogenous perturbations, which have largely focused on sensory integration for lost limbs, that now improve perception and task performance such as restoring biomimetic gait post amputation (Song et al. 2024). Beginning in the 1960s, peripheral invasive ANG recordings employing minimally invasive microneurography revealed peripheral skin sympathetic bursting and/or firing rates that are indicative of differential baroreflex activity in patients with and without arrythmia or cardiac disease (Vallbo et al. 2004). Equivalent decoding of interoceptive signaling of organ dysfunction and/or internal inflammation associated with infection and sick symptoms has recently gained traction within the neurotechnology community (Pavlov and Tracey 2017; Steinberg et al. 2016; Zanos et al. 2018; Bu et al. 2024; Bu et al. 2022) with a variety of potential use cases (Sammons et al. 2024). One such use case recently developed is focused on inflammation and infection decoding by the vagus nerve and sympathetic neuronal structures (Zanos et al. 2018; Bu et al. 2024; Bu et al. 2022; Zanos 2019). Traditionally, medicine has relied on diagnosing diseases based on symptoms like fever, chills, aches, mucus production, cough, and headache. Physiological biomarkers, such as pulse and blood pressure, are already used to evaluate the severity of infectious illnesses (Raith et al. 2017). Evolving on these physiological biomarkers, recent studies have shown that waveform data, including ECG (electrocardiogram), respirogram, and temperature can predict the severity of severe inflammation associated with sepsis (Wijk et al. 2023; Wickramaratne 2020; Kam and Kim 2017; Kam 2022; Shashikumar et al. 2021), and remarkably are able to predict specific pathogen infection (with high sensitivity and specificity) based on recorded time-series change in physiology (Farhang-Sardroodi 2022; Zaman et al. 2023; Vargas et al. 2022; Sawant et al. 2021; Yanamala et al. 2021; Cuesta-Frau et al. 2020). Beyond physiological metrics of infection, preclinical work demonstrates resting vagus nerve action potential recordings respond to inflammatory proteins. Using several different sensing modalities including cuff electrodes in anesthetized rats during inflammatory cytokine injection (Zanos et al. 2018), investigators detected ensemble action potentials and identified evoked compound action potentials upon stimulation (Metcalfe 2018). Moreover, recent work demonstrates that vagus nerve action potentials uniformly synchronize with the respiratory cycle in porcine models (Vallone et al. 2021). This neural effect was supported by two separate human microelectrode studies (Patros et al. 2022; Farmer 2024). Recent human clinical work also demonstrates that implanted gastric electrical stimulation results in measurable evoked potentials emanating from the vagus nerve when measured with surface electrodes, further opening the door to non-invasive vagus nerve monitoring (Ward et al. 2020). Other preclinical studies have measured (via cuff electrode) superior cervical ganglion activity with hypertensive stress tests, i.e., injection of adrenaline (Armour et al. 1998; Cassaglia et al. 2008) or during painful stimuli (McLachlan et al. 1997). These studies uniformly demonstrate immediate (within seconds) change in cervical sympathetic neuronal activity (superior cervical ganglion) with each challenge (Armour et al. 1998; Cassaglia et al. 2008; McLachlan et al. 1997).

Similarly, when the body starts responding to a pathogen, it is feasible to sense that initial response, possibly several hours before a patient becomes symptomatic. This is due to the fact that C-fibers, which comprise approximately 80% of the vagus afferent fibers, are known to respond to low levels of inflammatory proteins, i.e., cytokines as low as 5 pg/ml of TNF (Myers 1999). Our own work differentiates ventral cervical ANG activity beginning at relatively low levels enabling multiclass classifiers to distinguish 5 vs. 70 vs. 500 pg/ml of intravascular TNF (unpublished data). Preclinical work by Zanos et al. and others demonstrate both LPS and inflammatory cytokines elicit increases in afferent vagal (MacNeil et al. 1996; Hayden et al. 1998; Katayama et al. 2022) and afferent sympathetic nerve firing frequency (Katayama et al. 2022), within minutes of administration. In fact, recent work has shown that it is possible to isolate and decode LPS-induced and cytokine-specific vagus neural firing responses (i.e., specific for ensemble/cluster spikes derived from aggregate compound action potentials) (Zanos et al. 2018). This phenomenon was observed for IL-1β and TNF-α via cuff electrode recordings of ventral cervical vagal activity (Steinberg et al. 2016; Zanos 2019; Niijima et al. 1995; Niijima 1992; Niijima 1996; Silverman et al. 2018). TNF-α concentration was similarly correlated to the carotid sinus nerve firing activity (a branch of the sympathetic chain located in the ventral cervical carotid sheath), further highlighting that ventral cervical neurons are responsive to an inflammatory challenge (Katayama et al. 2022). Analogously, our own human work in which LPS is injected intravenously, confirmed a ventral cervical autonomic neurographic response that was cytokine specific (Bu et al. 2024). *Like prior work that is capable of classifying multiple different pathogens with recorded time series physiology data* (Vallone 2022; Zaman et al. 2023; Vargas et al. 2022; Sawant et al. 2021; Yanamala et al. 2021; Cuesta-Frau et al. 2020; Fernández et al. 2016; Alvarado et al. 2019; Huang et al. 2014*) in the near future, prediction of host pathogen infection by decoding ANG cytokine specific responses could be feasible.*

Further, in healthy subjects, ANG response to LPS injection segregated subjects into an endophenotype-specific high cytokine response and high neural signaling compared to a low cytokine response with lesser neural firing (Bu et al. 2024). Well-established immune cell disinhibition occurs in mental health with stress anxiety and depressive disorders in which the fight or flight responses are more common (Ravi et al. 2021; Michopoulos et al. 2017; Biltz et al. 2022). In turn, mental health disorder-mediated immune cell disinhibition culminates in hyperinflammatory responses when compared to healthy control subjects (Ravi et al. 2021; Michopoulos et al. 2017; Biltz et al. 2022; Lerman et al. 2016; Agorastos et al. 2019), while ANG may mirror this relationship. A better understanding of an individual’s neuroimmune axis, i.e., the relationship of mental health severity upon inflammation and resultant ANG is currently being verified by our group, in which in-vitro and in-vivo responsivity to LPS is correlated to the patient’s mental health disorder severity. Further, this information may be utilized to titrate and optimize anti-inflammatory therapies (either drug or neuromodulation based) in this population.

In conjunction with monitoring the autonomic signal, we can interpret the information of the body by seeing how the innate immune response proteins are being produced over time and to what extent. Pro- and anti-inflammatory processes begin promptly after sepsis onset; in general, there is a predominance of an initial hyperinflammatory phase, the magnitude of which is determined by several factors, including pathogen virulence, bacterial load, host genetic factors, and host comorbidities. The extreme diversity of the host human immune system forged and maintained throughout evolutionary history, has to date provided a potent defense against known and evolving opportunistic pathogens. Due to the perpetual genetic pressure across the millennia, host immunity is intrinsically heterogenous, because it is controlled by the most polymorphic genes and is shaped by highly sensitive environmental sensors (Liston et al. 2021; Wilk et al. 2021; Giroux 2020)*.* While most patients survive the initial hyperinflammatory phase of sepsis, some will not. If identified, hyperinflammatory patients with severe disease who are provided life-saving anti-inflammatory medications (steroids) demonstrate improved survival (Lyu et al. 2020); however, survival acutely depends on selecting endotypes deemed responsive to steroid therapy (Antcliffe et al. 2019), and can depend on indicators of innate and/or adaptive immune response (Yao et al. 2022). In contrast, some patients will enter a more protracted phase of sepsis characterized by increasing immunosuppression. Multiple lines of evidence demonstrate that during the protracted immunosuppression phase, patients may develop severe depletion of immune effector cells that compromise host immune defenses. Clinicians following standard of care broad-spectrum antibiotic treatments and aggressive source control measures may still identify patients with: 1) infections that are not eradicated, and/or 2) secondary host or hospital-acquired infections. Although highly promising, few host-directed immune cell stimulant therapeutics have been shown efficacious in human sepsis trials (Skrupky et al. 2011; Meisel et al. 2009) wherein the study participants were enriched by selecting hypo-inflammatory phase (immunosuppressed) participants. In both cases, successful treatment of hyperimmune or the immunoparalysis state was highly dependent on the enriched population known to respond to the respective therapy. Fitting to this construct, time series physiological based measures and ANG can be deployed to continuously calculate an individual’s endotype i.e., hyper or hypo-immune response. In the future, ANG could detect low-grade infections and their consequences (e.g., one theory of “long COVID” is that a persistent inflammatory response, triggered by residual viral material and/or active virions, produces persistent fatigue and respiratory distress) (Talla et al. 2023). Intriguingly, microelectrode recordings from patients with Chronic Obstructive Pulmonary Disorder (COPD) known to have autonomic dysregulation and peripheral hyperinflammation are being studied by Speisshoefer and colleagues (Spiesshoefer et al. 2022); the microelectrode recordings may stratify disease severity, peripheral inflammation, and response to a therapeutic drug. Beyond the peripheral organ disorders, there are also numerous brain disorders driven by or maintained due to an elevated and persistent inflammatory state; these disorders include tauopathies, epilepsy, Parkinson’s disease, pain, tinnitus, while brain inflammation is now considered to be contributory to mental health disorders and many other health disorders broadly prevalent in our society (Ravi et al. 2021; Michopoulos et al. 2017; Langworth-Green et al. 2023; Searchfield 2021; Sampson et al. 2016; Mulak and Bonaz 2015; Katrinli et al. 2022). Vagus neuronal tracts are now understood to be a conduit for pathological proteins such as alpha-synuclein transmitted from the gut to the brain causative of Parkinson's disease phenotype (Zhang et al. 2023; Kluge 2024; Kim et al. 2019). To better understand this relationship, our group is currently investigating whether or not ANG is altered in this population pre-to-post transcutaneous cervical VNS (tcVNS) with the primary outcome aimed at optimizing the treatment of gastroparesis in affected Parkinson’s disease patients. Although non-invasive ANG monitoring seemingly presents many opportunities to objectively stratify an individual’s disease state across time, multiple challenges will need to be addressed to reach a future in which closed-loop autonomic neuromodulation is achieved. Namely, robust linkage of ANG to diverse disease states remains to be identified, while signal drift and signal loss is likely to contribute to complexity in reading ANG signals for each individual especially as their disease progresses. To complement physiological monitoring, innovations are also emerging for enabling real-time or frequent molecular monitoring, such as for glucose, cytokines and hormones, which can further contribute to stratifying individual’s disease states and tracking of treatment outcomes (Vignesh et al. 2024; Saha et al. 2023; Li et al. 2023).

#### Invasive and emerging non-invasive autonomic therapeutics – stimulation

Parallel to diagnostics, we’re seeing the advent of therapeutics. Variability in patient outcome and disease progression can be tied to the explicit health of the individual patient. Beyond the genetics that contribute to the expression of inflammatory proteins for a particular patient, there is the health of their immune system and neuro-immune axis, which can change due to environmental factors over time (Biltz et al. 2022; Katrinli et al. 2022). The inflammatory reflex, a neural circuit that regulates immune responses, can be enhanced through electrical stimulation of the vagus nerve anti-inflammatory reflex that activate the efferent arc of the vagus nerve culminating in activation of splenic mediated inhibition of cytokine release in trafficking immune cells (Koopman et al. 2016a; Pavlov and Tracey 2017; Andersson and Tracey 2012; Borovikova et al. 2000). Enhanced efferent signaling by minimally invasive implanted vagus nerve stimulation (miVNS) has been shown to significantly reduce cytokine production and attenuate disease severity across a range of experimental models. These include preclinical animal models of endotoxemia (Andersson and Tracey 2012; Borovikova et al. 2000; Rosas-Ballina et al. 2011), colitis (Meregnani et al. 2011), and various other inflammatory syndromes (Maanen et al. 2009). This body of evidence underscores the potential of vagus nerve stimulation as a therapeutic approach for modulating inflammatory responses in diverse pathological conditions. Clinical miVNS has emerged as a promising treatment for various inflammatory conditions in which the normal neuroimmune axis regulation of inflammation has become dysregulated. Koopman et al. (2016a) demonstrated that cervical miVNS can inhibit cytokine production and attenuate disease severity in rheumatoid arthritis (RA) patients, while Bonaz et al. (2016) showed its efficacy in inducing clinical remission in 5 of 7 patient’s Crohn's disease (CD) (Bonaz et al. 2016; Sinniger et al. 2020). In both cases of RA and CD, autonomic tone measured by HRV predicted disease severity (Koopman et al. 2016b) and/or likelihood of response to the miVNS (Bonaz et al. 2016; Sinniger et al. 2020). Transcutaneous auricular VNS (taVNS) and transcutaneous cervical (tcVNS) offer a non-invasive alternative, with Yap et al. (2020) reviewing its anti-inflammatory effects in conditions such as sepsis, stroke, and depression (Yap et al. 2020). Intriguingly, auricular vibratory VNS has also shown promise in modulating whole blood LPS stimulated inflammatory cytokine concentrations, and clinical DAS28-CRP scores as demonstrated by Addorisio et al. (2019) in their study on RA patients (Addorisio et al. 2019). While meta-analyses can help distinguish specific neuroimmune axis regulation effects for specific cytokines across miVNS, taVNS, and tcVNS, there still remains high variability in outcomes, in part due to varying cytokine measurement technologies deployed (i.e., peripheral blood vs. whole blood cultured with and without LPS) that contribute to the observed differences that may obscure purported device specific variability (Melo 2024).

Therapeutic non-invasive studies performed by separate research teams led by Drs. Hubert Lim and Chris Puleo through the DARPA ElectRx Program discovered that one can modulate the neuroimmune axis by applying external ultrasound to the spleen for treatment of inflammatory arthritis or an LPS-induced response (Cotero et al. 2019; Zachs et al. 2019). If activated, the innate immune response can result in immediate, within minutes, cytokine production and inflammation triggering further immune cell activation and regulation responses. An afferent to efferent vagus anti-inflammatory reflex (the cholinergic anti-inflammatory pathway (CAP)) may be endogenously activated (depending on the strength and duration of the inflammatory insult) and throttle down the inflammatory response, preventing runaway systemic inflammation; it may occur on a short or long timescale depending on the health condition (e.g., sepsis or chronic disease inflammation, respectively). But, for some patients, the anti-inflammatory response is insufficient. By targeting the efferent nerves and immune cells in the spleen with focused ultrasound, the anti-inflammatory response can be directly modulated. Splenic focused ultrasound stimulation (sFUS) (Zanos et al. 2023; Graham 2020), and more generally CAP activation, which is also being studied with splenic neurovascular bundle stimulation (Brinkman et al. 2022), shows great promise in early clinical trials for treating inflammatory disorders such as rheumatoid arthritis (Koopman et al. 2016a; Lim 2024), and a recent large trial completed by SetPoint Medical (Yakov 2024) with further opportunities for Crohn’s disease (Sinniger et al. 2020; D’Haens et al. 2023) treatment that are now underway. Our group is also actively exploring treatment models for acute infectious hyperinflammation, such as the human intravascular LPS injection challenge, with therapeutic intervention using sFUS.

Recently, preclinical studies have demonstrated successful treatment of myocarditis or pulmonary hypertension in response to sFUS involving an anti-inflammatory mechanism (Zafeiropoulos et al. 2024; Liu et al. 2023). In inflammatory or reactive airway diseases, such as asthma and Chronic Obstructive Pulmonary Disease (COPD), leveraging the anti-inflammatory effects of sFUS may also reduce respiratory tract inflammation, thereby decreasing eosinophil accumulation in respiratory tract tissue pathognomonic to asthma and COPD exacerbations.

For autonomic neural stimulation with miVNS, tcVNS, taVNS, and ultrasound application, the hypothesis is that the underlying treatment is, at least in part, acting on or superseding the autonomic set point. Beyond autonomic tone, the ANS and its brain networks, i.e., the central autonomic network (CAN) exhibits an underlying set point that may be influencing dysregulated autonomic tone (Thayer and Fischer 2009; Sie et al. 2019; Howard et al. 2024; Lerman et al. 2019; Valenza et al. 2024; Ma et al. 2024). Dysregulated autonomic tone is multifaceted and not necessarily mutually exclusive; for example in patients with heart failure, carotid nerve stimulation of the baroreceptors in the carotid sinus modulates autonomic balance to improve cardiovascular function. Zile et al. (2020) reported on the results of the BeAT-HF trial, which demonstrated that baroreflex activation therapy significantly reduced sympathetic activity, increased parasympathetic activity, and improved quality of life, exercise capacity, and NT-proBNP levels in heart failure patients (Zile et al. 2020). This landmark FDA approval of the CVRx Barostim Neo system in 2019 represents a major step forward in the clinical application of invasive surgically implanted autonomic modulation for cardiovascular disorders, especially as multiple prior miVNS trials were unsuccessful (Ferrari et al. 2017; Gold et al. 2016); it potentially points to limitations of acute VNS in cardiac disease. While baroreflex activation clinical efficacy was proven, changes in cardiac neural network targets engaged and respective brain central autonomic networks (CANs) remain to be determined (Herring 2024). Highly co-morbid with cardiovascular disease, posttraumatic stress disorder (PTSD) consistently demonstrates hyper sympathetic tone as measured by HRV metrics (high frequency-HRV) prior to and after (Minassian et al. 2016; Minassian n.d.) the traumatic event (Stout et al. 2022). Intriguingly tcVNS significantly reduced sympathetic tone in Veterans with PTSD (Gurel et al. 2020). Especially in PTSD, hyperactive salience and anticipatory neural networks are activated in response to aversive stimuli, and they remain active to a greater extent than in healthy controls (Zhu et al. 2023; Simmons et al. 2004; Menon 2011). These hyperactive salience and anticipation network nodes in turn directly activate CAN nodes, resulting in the hyper-sympathetic response further perpetuating a fight-or-flight response that over time desensitizes immune cells to peripheral anti-inflammatory signaling (Michopoulos et al. 2017; Katrinli et al. 2022; Minassian et al. 2016; Stout et al. 2022). A non-invasive approach to autonomic neuromodulation that lowers sympathetic tone may directly or indirectly influence immune regulation and inflammation. Ongoing and planned clinical trials will determine whether long-term VNS impacts immune balance and inflammatory pathways in diseases where chronification of inflammation plays a significant role in disease progression (e.g., for cardiac disease, ulcerative colitis (UC), CD, COPD, pulmonary hypertension, and RA).

More recently, the prospect of an advanced role of the neuroimmune axis in maintaining immune homeostasis has been proposed. That is, several groups have uncovered clusters of neurons within the brainstem, which when activated by afferent nerve signals control efferent nerve signaling back down to peripheral organs and anti-inflammatory pathways (Kressel et al. 2020; Jin et al. 2024). An elegant series of experiments using the targeted recombination in active populations (TRAP) system to target and map neurons activated by specific inflammatory mediators has further revealed that distinct populations of vagal neurons respond to pro- versus anti-inflammatory cytokines, and that this information is conveyed to a body of neurons within the nucleus of the solitary tract (Jin et al. 2024). Importantly, it was further shown that removing signaling in this body-brain neuroimmune circuit during inflammatory challenge resulted in unregulated and out of control immune response. These experiments now reveal that neuroimmune pathways may mediate both positive- and negative-feedback modulation of immune cells, and further advances the notion of the existence of a biological rheostat within the central nervous system (CNS) controlling the timing and magnitude of the peripheral inflammatory response. Our group has carried out brain MEG studies on individuals pre-to-post intravenous LPS injection, (unpublished data) that demonstrate cholinergic nuclei responsivity to the LPS challenge. Work is under way to stratify identified MEG based change in cholinergic nuclei response and LPS induced cytokine concentration change.

However, despite these recent advances, much remains unknown. The specific efferent nerve circuits that are modulated by the integrative neurons within the brainstem and CNS are unknown. Beyond the splenic cholinergic anti-inflammatory pathway, several other neuroimmune reflexes have been identified that may be activated by these neurons to maintain homeostasis, including a vagal-adrenal pathway (e.g., releasing dopamine as an anti-inflammatory mediator) (Torres-Rosas et al. 2014), an intestinal cholinergic anti-inflammatory pathway (that may act independent of splenic CAP) (Goverse et al. 2016), and lymphatic-neural pathways (that may control immune cell distribution) (Cotero et al. 2020). The extent to which the central nervous system mediators can first detect specific anatomical and pathogen specific signals of infection, and then choose among different efferent anti-inflammatory responses remains a major topic of investigation. Perhaps more intriguing, several recent investigations have revealed that neuroimmune pathways may also effect adaptive immune responses, including the maturation and antibody-response of splenic plasma cells (Cotero et al. 2020). This nerve mediated response was shown to depend on both vagal-mediated nerve signals and the activity of the hypothalamic–pituitary–adrenal (HPA) axis, demonstrating yet another potential integrative function of the collective body-brain neuroimmune axis.

#### What we’re working toward

Non-invasive autonomic bioelectronic medicine is on the cusp of revolutionary changes in healthcare. Understanding how the body functions holistically and regulating the body to fight off infection and illness is already a shift in understanding how to diagnose and treat a patient. Beyond the immediate change in care of the individual patient, new non-invasive closed loop autonomic neuromodulation technologies have potential significant near term second order effects beyond the individual patient.

#### Special use case of personalized closed loop autonomic neuromodulation diagnostics and therapeutics

As an exemplar application in which human/host sensing is intertwined, both pathogen and host respond to each other, and they do so in varied but predictable ways; thus, physiological and ANG measures charting in how a pathogen attacks and how the body responds can inform the clinician of which pathogen a patient is responding to (Bu et al. 2024; Bu et al. 2022; Sammons et al. 2024; Zanos 2019; Raith et al. 2017; Wijk et al. 2023). Knowing which cytokines ramp up and how they ramp up will create a fingerprint that details which pathogen a patient is responding to before the patient may even be symptomatic (Mann 2018; McClain et al. 2016; Nguyen-Van-Tam et al. 2020). Combining data from autonomic responses with molecularly derived blood or saliva samples could potentially allow pathogen diagnosis (Bu et al. 2024; Farhang-Sardroodi 2022; Zaman et al. 2023; Vargas et al. 2022; Sawant et al. 2021; Yanamala et al. 2021; Cuesta-Frau et al. 2020; Mann 2018; McClain et al. 2016; Nguyen-Van-Tam et al. 2020) before traditional techniques would even know a patient is sick; this information may trigger an at home (or in a clinical environment) confirmatory blood or saliva sample to diagnose the disease.

Being able to diagnose a pathogen rapidly and effectively may help bring an end to, or at least slow the progression of, antibiotic resistance by improving antibiotic stewardship. Instead of using broad-spectrum antibiotics to treat a patient, not knowing what they are sick with, we can tailor treatment to the patient and the pathogen. With host-based pathogen agnostic diagnostic time series-based physiological models (Farhang-Sardroodi 2022; Zaman et al. 2023; Vargas et al. 2022; Sawant et al. 2021; Yanamala et al. 2021; Cuesta-Frau et al. 2020) concatenated to ANG features (Zanos et al. 2018; Bu et al. 2024; Bu et al. 2022; Zanos 2019), future models may predict specific pathogen infection and instantly notify the clinician of what the patient is infected with at a basic level, bacteria versus others (i.e., virus or non-bacterial infection). Refined models may further improve when select features are added from the molecular (Rao et al. 2022) and/or physiological parameter space (Farhang-Sardroodi 2022; Zaman et al. 2023; Vargas et al. 2022; Sawant et al. 2021; Yanamala et al. 2021; Cuesta-Frau et al. 2020). These host-based pathogen agnostic diagnostics are rapidly gaining traction with expected clinical approved use in the near future, while further refined models may stratify severity associated with each infection (Antcliffe et al. 2019; Yao et al. 2022; Balch et al. 2023). With these models in hand, the clinician may hone their approach upfront by understanding the individual’s molecular and autonomic subtype, i.e., how that particular patient will respond to a particular pathogen and what treatments are most likely to benefit that individual patient (Katayama et al. 2022; Niijima et al. 1995). Folding in direct relationships with the patients autonomic set point, the clinician can begin to fine-tune the patient’s immune system via titrated neuromodulation or end-organ stimulation, in which the therapy may be life saving for certain patients (Zanos et al. 2023; Graham 2020; Tynan et al. 2024; Kurata-Sato et al. 2024). If this patient-centric approach is not enough to overcome the infection, broad spectrum antibiotics may instead be used as a last resort instead of as a first line therapeutic.

Another potential benefit is being able to reduce large scale disease transmission. In the recent pandemic, we hoped that infected individuals would not fly through crowded airports, but this is hindered by at least two significant problems. One, patients may not yet be symptomatic. Two, patients may be symptomatic, but not realize that they have an infectious pathogen that can easily spread in such environments. These host-based pathogen agnostic diagnostics made available by newly developed autonomic sensing allows pre-symptomatic diagnosis, potentially cutting down on problem one. Fingerprinting of diseases following the patient response allows for advanced assessment of problem two.

Beyond the individual level, witnessing new or unexpected host-based time series autonomic responses can indicate discovery of a new pathogen (Zaman et al. 2023; Vargas et al. 2022; Sawant et al. 2021; Cuesta-Frau et al. 2020; Fernández et al. 2016; Alvarado et al. 2019; Huang et al. 2014). This capability would enable advanced early detection of new pathogens, ideally before the pathogen can spread to endemic or pandemic levels. Generally, these pandemic preparatory diagnostic capabilities will allow for better health outcomes on both the individual and societal scale.

#### Closed-loop neuromodulation

Reframing our understanding of the body and disease, some conditions aren’t just the result of a pathogen or a genetic defect but a shift in how the body maintains itself through homeostatic mechanisms (Sammons et al. 2024). The autonomic set point inclusive of both the CAN and vagus anti-inflammatory reflex that regulates the neural circuit governing inflammation in the body may be predisposed to shift based on genetic factors and may be induced to shift based on environmental factors within and across generations (Shrira et al. 2017). While current pharmaceutical treatments may target the output of the CAP (e.g., an anti-TNF drug), autonomic closed loop bioelectronic medicine may instead focus on shifting the autonomic set point to fully alleviate the condition, such as for autoimmune disorders (e.g., rheumatoid arthritis and inflammatory bowel disease) as well as many brain disorders (e.g., epilepsy, tauopathies, pain, etc.). Evidence already exists that the autonomic set point is plastic and modifiable; recent work from both Fullerton and Lehrer demonstrates that pre-intravenous LPS injection cold water exposure and deep breathing exercises significantly modify post-intravenous LPS injection autonomic tone change and cytokine release (Fullerton 2016; Lehrer et al. 2010).

Like advances discussed in closed loop DBS and BCI aimed to treat Parkinson’s Disease, epilepsy, and SCI, the treatment of chronic pain is increasingly understood. Using the gate control theory of pain, bioelectronic spinal cord stimulation or peripheral painful nerve-based stimulation, paresthesia induced pain relief started as a means of closing the gate on more significant pain. However, as clinical practice has advanced and basic science is catching up, there are several modes of chronic pain treatment with spinal cord stimulation. Paresthesia, non-paresthesia, high/low/ultra-low frequency, and burst spinal cord stimulation all offer means of relief for patients with chronic pain (Caylor et al. 2019). Instead of simply closing a gate, there is a multifaceted interplay of A-delta, A-beta, C fibers, and their modulators that either inhibit pain signaling or reduce the promotion of pain signaling (Caylor et al. 2019). Newer spinal cord stimulation device technologies are now FDA approved using closed loop evoked potential based spinal cord stimulation demonstrating efficaciousness in pain populations (Mekhail et al. 2022; Vallejo et al. 2021). These systems are now being investigated for additional use case scenarios including to reduce respiratory tract infections, improve bowel management in patients with neurological impairment, and complement stroke rehabilitation either with (Powell et al. 2023), or without device implantation (Moritz 2024). Beyond spinal cord stimulation, peripheral neuromodulation methods could also alter systemic inflammatory or autonomic responses that promote pain and pain chronification. Research by Koopman et al. (2016a) and Bonaz et al. (2016) both point to the fact that an individual’s autonomic set point contributes to the variance observed pre-to-post the autonomic neuromodulation (Bonaz et al. 2016; Sinniger et al. 2020; Koopman et al. 2016b).

**Fig. 2:**
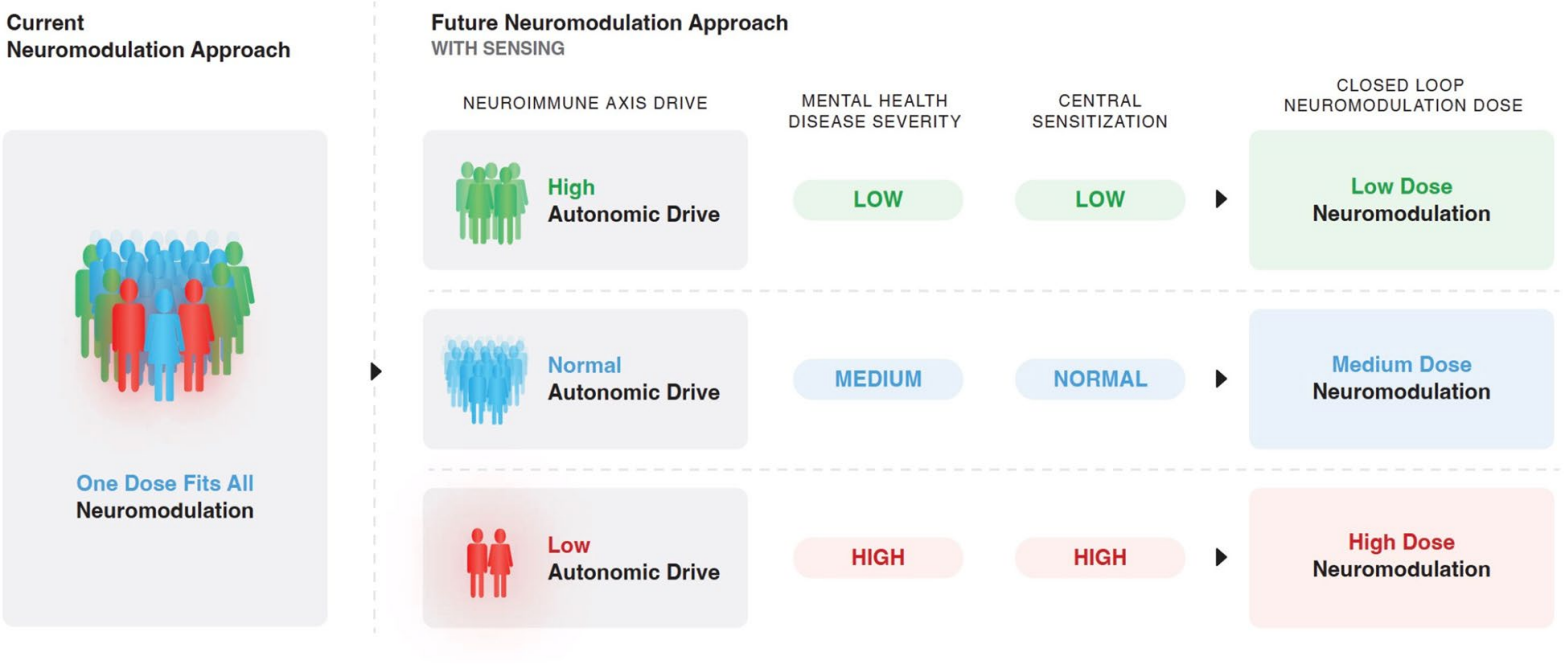
Current neuromodulation instantiates a one dose fits all approach. In the near future, personalized medicine employing sensing platforms may identify fingerprints of biomarkers. They will include inflammatory pathways, neuroimmune axis, and/or central sensitization (common in chronic pain syndromes) as bioindicators useful in titration of the therapeutic stimulation paradigm, such as with electrical stimulation or ultrasound stimulation of neural and non-neural cells. Titrated therapeutic stimulation may span dosage duration and/or frequency of dosage during a circadian cycle. Closed loop stimulation titrated in real time can provide an optimized density of therapeutic energy preventing neuroplasticity induced adaptation

In a recent translational preclinical sFUS study by Zanos et al. (2023), large effect sizes were observed in the inbred Sprague–Dawley rats with a significant reduction in LPS spiked whole blood cultured cytokine concentrations. In a pilot clinical trial, splenic ultrasound insonification was also shown to activate an anti-inflammatory response in healthy human subjects, and the magnitude of this biological effect, measured by whole blood TNF production in response to ex vivo LPS was comparable to that previously reported by activation of the neuroimmune pathway at the spleen using invasive vagus nerve stimulation. However, the potential for target site and stimulation parameter specific effects will require additional studies to understand dosing in light of the observed heterogenous response in human populations (Zanos et al. 2023). Analogously, our own group observed heterogenous human inflammatory responses to LPS, both in-vivo (Bu et al. 2024) and ex-vivo (Lerman et al. 2016), suggesting that autonomic set points are distinguishable even amongst healthy individuals. *To address this inherent heterogeneity, it is the authors belief that both invasive and non-invasive autonomic neuromodulation must develop closed loop device technologies aimed to titrate the specific dose needed over time* (Fig. 2)*.*

While ultrasound neuromodulation has the potential to provide targeted and anatomically precise stimulation (Sinniger et al. 2020; Cotero et al. 2022a; Cotero 2022; O’Reilly 2024), it has been shown to activate both efferent and afferent neural pathways, inevitably adding complexity in closed loop control system design. Furthermore, there is growing evidence that one mechanism of activation relies on distinct stimulation of ultrasound-sensitive ion channels (Cotero et al. 2022a; 2022; O’Reilly 2024). Thus, additional knowledge on the expression patterns of ultrasound sensitive ion channels in discrete populations of neurons, including activation of end-organ receptors or feedback signaling pathways to the brain involved in neuroimmune circuits, may be required for further honing ultrasound-specific closed-loop systems. More specific mapping and understanding of the overlap between sub-types of neural and non-neural cells conveying immune-specific information, and those targeted and activated by specific stimulation technologies will be required for future design of closed-loop neuromodulation systems.

Considering sepsis, the understanding and treatment have evolved significantly in the last 40 years. Historically, sepsis was seen primarily as a consequence of infection by bacteria, viruses, or other pathogens, with the focus on the body being "overwhelmed" by these infectious agents. This perspective emphasizes the role of the pathogen in causing the disease state, with treatments largely focused on eradicating the infectious agent(s) using antimicrobials. However, recent research has illuminated the complexity of sepsis, revealing it as a dysregulated host-specific response involving both the innate and adaptive immune systems (Maslove et al. 2022). The current understanding is that sepsis can result from a hyperactive innate immune response, which can cause widespread inflammation and tissue damage, and/or a subsequent insufficient adaptive immune response, which fails to effectively control the infection and restore homeostasis. The next evolution in this understanding is to develop tools that predict in real time these divergent immune responses, thereby better informing the clinician of the patient treatment needs. Being able to monitor the autonomic nervous system and neuronal signaling from cytokine induced compound action potentials allows for diagnosing both a hyperactive innate immune response as well as a suppressed adaptive immune response. Knowing a patient is experiencing a hyperactive innate immune response, they can be treated with a bioelectronic medicine like sFUS to suppress the inflammatory effect, as opposed to corticosteroids, that may increase 28-day mortality in patients with an immunosuppressed response to sepsis (Dong et al. 2023). Meanwhile, knowing that a patient is experiencing a stalled or paralyzed adaptive immune response, patients can receive a host directed therapeutic like GM-CSF or other drugs in development to stimulate their underactive immune system (Skrupky et al. 2011; Meisel et al. 2009; Pflaumer 2022).

## Conclusion

**Fig. 3:**
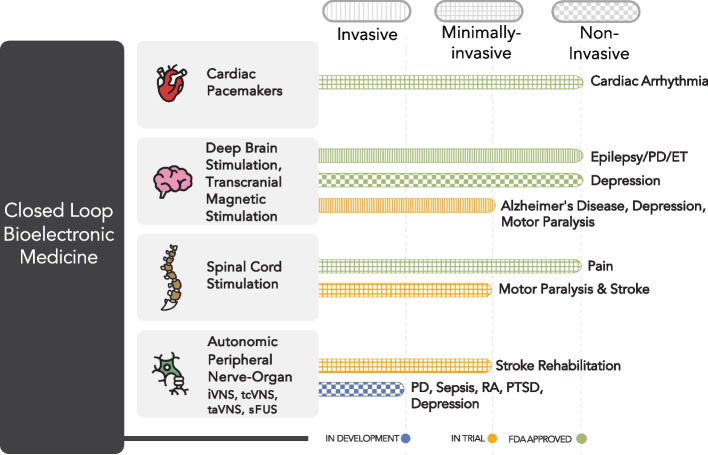
Current FDA approved closed loop devices include cardiac pacemakers, spinal cord stimulation (SCS) for pain, and closed-loop deep brain stimulation for epilepsy, Parkinson’s Disease (PD), and Essential Tremor (ET) and non-invasive closed-loop transcranial magnetic stimulation (TMS) for Depression (blue line: in development; light orange line: in trial; light green line: FDA approved). Clinical trials are currently underway testing efficacy of: closed loop SCS for stroke and motor paralysis, closed loop deep brain stimulation for depression and Alzheimer’s disease. Devices are in development to provide minimally and noninvasive closed loop autonomic neuromodulation. Invasive: vertical lined bar; minimally invasive: cross-hatched bar; and non-invasive: checkered bar. miVNS: minimally invasive VNS; tcVNS: transcutaneous cervical vagus nerve stimulation; taVNS: transauricular VNS; sFUS: focused ultrasound stimulation

The historical trajectory of bioelectronic medicine, from the rudimentary applications of electric fish in antiquity to the sophisticated invasive technologies of the twentieth century, has now reached a pivotal inflection point with the advent of non-invasive closed-loop neuromodulation. As the field transitions from invasive techniques such as deep brain stimulation and vagus nerve stimulation to non-invasive approaches such as focused ultrasound and magnetic stimulation, we are witnessing the confluence of technological advancements and clinical practicality for improving access to these technologies. Non-invasive methods, including recordings with EEG, MEG, and ANG, and stimulation with tools such as TMS and focused ultrasound, are powerful tools capable of modulating the central and peripheral nervous system, both of which can alter immune and autonomic function without the need for surgical intervention (Fig. 3).

The promise of these next-generation non-invasive systems lies not only in their ability to mitigate the risks associated with invasive procedures but also in their capacity for real-time, individualized therapy. Closed-loop systems continuously monitor key physiological markers and autonomic signals, enabling precise, dynamic adjustments tailored to the patient’s specific needs. This personalized approach marks a fundamental shift in the treatment paradigm, allowing therapies to be optimized in real-time based on individual responses rather than static protocols. The shift from invasive to non-invasive technologies represents a significant and equitable leap forward in bioelectronic medicine, positioning it at the forefront of precision healthcare, where therapies are designed to be as adaptable and responsive as the conditions they seek to treat.

As we consider the future of medical treatment, the promise of non-invasive closed loop neuromodulation represents a sustainable, equitable, versatile, and efficient alternative to conventional pharmaceuticals. It offers the potential to maintain and restore health with significantly reduced dependence on chemical and biological resources. In light of these advantages, the exploration and development of host-directed closed loop therapeutics not only promise to revolutionize healthcare delivery but also to expand our capacity for providing care in diverse and challenging environments and enabling more equitable and accessible healthcare for all.

## Data Availability

No datasets were generated or analysed during the current study.

## Abbreviations

ANS: Autonomic Nervous System
ANG: Autonomic Neurography
BCI: Brain Computer Interface
CAN: Central Autonomic Network
CAP: Cholinergic Anti-inflammatory Pathway
CNS: Central Nervous System
COPD: Chronic Obstructive Pulmonary Disease
CD: Chron’s Disease
DBS: Deep Brain Stimulation
EEG: Electroencephalography
ElectRx: Electrical Prescriptions
MEG: Magnetoencephalography
DARPA: Defense Advanced Projects Agency
ECG: Electrocardiogram
ET: Essential Tremor
ECAP: Evoked Compound Action Potential
FDA: Federal Drug Agency
HRV: Heart Rate Variability
HPA axis: Hypothalamic-Pituitary-Adrenal Axis
IL-1β: Interleukin 1 Beta
LPS: Lipopolysaccharide
miVNS: Minimally Invasive VNS
N^3^ program: Next-Generation Nonsurgical Neurotechnology
NIH: National Institutes of Health
PD: Parkinson’s Disease
PNS: Peripheral Nervous System
RE-NET: Reliable Neural-Interface Technology
SCS: Spinal Cord Stimulation
sFUS: Splenic focused ultrasound
SPARC: Stimulating Peripheral Activity to Relieve Conditions
TNF-α: Tumor Necrosis Factor Alpha
TMS: Transcranial Magnetic Stimulation
tcVNS: Transcutaneous Cervical VNS
taVNS: Transauricular VNS
TRAP: Targeted Recombination In Active Populations
UC: Ulcerative Colitis
VNS: Vagus Nerve Stimulation
